# The impact of comorbid anxiety on quantitative EEG heterogeneity in children with attention-deficit/hyperactivity disorder

**DOI:** 10.3389/fpsyt.2023.1190713

**Published:** 2023-07-12

**Authors:** Changwon Jang, Soowhan Oh, Hyerin Lee, Junho Lee, Inmok Song, Yerin Park, Eunji Lee, Yoo-Sook Joung

**Affiliations:** ^1^Department of Psychiatry, Samsung Medical Center, Sungkyunkwan University, Seoul, Republic of Korea; ^2^Department of Psychiatry, Changwon Samsung Hospital, Changwon-si, Gyengsangnam-do, Republic of Korea

**Keywords:** quantitative electroencephalography, ADHD, anxiety, theta/beta ratio, cluster analysis

## Abstract

**Objective:**

The objective of this study was to compare quantitative electroencephalography (Q-EEG) characteristics of children with Attention-deficit/hyperactivity disorder (ADHD), taking into account the presence of a comorbidity for anxiety disorder. It also sought to investigate the impact of comorbid anxiety on the Q-EEG heterogeneity of children with ADHD.

**Method:**

A total of 141 children with ADHD but without comorbid anxiety (ADHD-Only), 25 children with a comorbidity for anxiety disorder (ADHD-ANX) and 43 children in the control group were assessed. To compare Q-EEG characteristics between groups, we performed ANCOVA (Analysis of Covariance) on relative power and theta/beta ratio (TBR) controlling for covariates such as age, sex, and FSIQ. Relative power values from 19 electrodes were averaged for three regions (frontal, central and posterior). Furthermore, cluster analysis (Ward’s method) using the squared Euclidian distance was conducted on participants with ADHD to explore the impact of anxiety on the heterogeneity of Q-EEG characteristics in ADHD.

**Results:**

There were no significant group differences in cognitive and behavioral measures. However, significant differences between groups were observed in the theta values in the central region, and the beta values in the frontal, central and posterior regions. In *post hoc* analyses, It was found that the ADHD-ANX group has significantly higher beta power values than the ADHD-Only group in all regions. For the theta/beta ratio, the ADHD-Only group had significantly higher values than the ADHD-ANX group in frontal, central and posterior regions. However, the control group did not show significant differences compared to both the ADHD-Only and ADHD-ANX group. Through clustering analysis, the participants in the ADHD-Only and ADHD-ANX groups were classified into four clusters. The ratios of children with comorbidities for anxiety disorder within each cluster were significantly different (*χ*^2^ = 10.018, *p* = 0.019).

**Conclusion:**

Attention-deficit/hyperactivity disorder children with comorbid anxiety disorder showed lower theta power in the central region, higher beta power in all regions and lower TBR in all regions compared to those without comorbid anxiety disorder. The ratios of children with comorbidities for anxiety disorder within each cluster were significantly different.

## Introduction

Attention-deficit/hyperactivity disorder (ADHD) is a neuropsychiatric disorder characterized by developmentally an inappropriate level of inattention, hyperactivity, and impulsivity. It is one of the most prevalent psychiatric disorders in childhood. Estimates of ADHD prevalence are highly variable, but it has been estimated that ADHD affects about 7.4% ∼ 9.5% of the childhood population (1–3).

Attention-deficit/hyperactivity disorder often accompanies psychiatric comorbidities, which not only complicates its differential diagnosis but also contributes to the heterogeneity of clinical features in children with ADHD (4). Anxiety disorder is one of the most common comorbid psychiatric disorders of ADHD (5). Given that anxiety can interact with symptoms of ADHD, such as impulsivity and hyperactivity (6), It is important to understand the biological and symptomatic interactions between ADHD and anxiety.

Previous studies have primarily focused on examining the cognitive and behavioral characteristics of children diagnosed with both ADHD and anxiety disorder. These studies have reported deficits in working memory (7), slower motor speed, and higher behavioral inhibition in children with ADHD (4, 8). However, To our knowledge, no study has focused on biomarkers that reflect the physiological characteristics of children with both ADHD and anxiety disorder.

Attention-deficit/hyperactivity disorder showed heterogeneity not just in its symptoms, but also in its brain activity, which can be examined by analyzing quantitative electroencephalography (Q-EEG) patterns. Q-EEG has been used to study neurophysiological basis of ADHD in the research field. It has also been used for assessing ADHD in the clinical field. Increased power in the theta band and decreased power in the beta band are the most consistently reported findings in ADHD children (9). Based on these findings, some studies have proposed that an excessive theta to beta ratio (TBR) has a diagnostic value for ADHD (10). However, a recent meta-analysis has concluded that Q-EEG characteristics of ADHD children are significantly heterogeneous. Therefore, the excessive TBR cannot be regarded as a reliable diagnostic measure for all individuals with ADHD (11).

Some studies have proposed that the heterogeneity within ADHD populations can lead to the classification of Q-EEG results into different subtypes, which may explain the conflicting findings observed in previous studies (12). Specifically, subtypes characterized by increased fast waves could potentially undermine the diagnostic value of TBR. Other studies have attempted to explore the relationship between QEEG heterogeneity and comorbidity within ADHD groups by comparing the clinical characteristics of each subgroup (13). However, the current pool of research in this domain remains insufficient. Given that anxiety disorder is one of the most common psychiatric disorders accompanied by ADHD and that anxiety disorder has been reported to have unique specific EEG characteristics such as excessive beta power at frontal, parietal and central regions (14, 15), further exploration of Q-EEG characteristics in children with ADHD who also have comorbid anxiety disorder is necessary to broaden our understanding of Q-EEG heterogeneity and the characteristics of ADHD.

Therefore, the goal of this study was comparing the QEEG characteristics of children with ADHD based on the presence and absence of comorbid anxiety. Additionally, we aimed to investigate whether the presence of comorbid anxiety disorder contributes to Q-EEG heterogeneity observed in children with ADHD.

## Materials and methods

### Subjects

The ADHD only group (ADHD-Only) and the ADHD with a comorbidity for anxiety disorder group (ADHD + ANX) included children who made their first visit to the Department of Psychiatry at Samsung Medical Center (Seoul, Republic of Korea) from 2017 to 2021.

The inclusion criteria for children with ADHD were as follows: (1) aged 6–13 years, and (2) diagnosed with ADHD (combined presentation, predominantly inattentive presentation, or predominantly hyperactive/impulsive presentation) according to the DSM-5 (the Diagnostic and Statistical Manul of Mental disorders, Fifth Edition) classification (16), based on clinical interview and the Kiddle Schedule for Affective Disorders and Schizophrenia for School-Age Children-Present and Lifetime Version (K-SADS-PL). Among the children with ADHD, those diagnosed with any anxiety disorder according to the DSM-5classification, based on clinical interview and K-SADS-PL, were assigned to the ADHD-ANX group, while those without comorbid anxiety disorder were assigned to the ADHD-only group. A healthy control group (HC) consisted of children aged 6 to 13 years who were recruited separately for comparison with the patient group between 2019 and 2020. All patients in the HC group had no psychiatric diagnosis based on the K-SADS-PL.

The exclusion criteria for all groups were as follow: (1) individuals with an IQ score lower than 70, as assessed by the Korean version of the Wechsler Intelligence Scale for Children 4th edition (K-WISC-IV); (2) individuals with current episodes of major depressive episode or manic episode based on clinical interview or the K-SADS-PL; (3) individuals with a history of psychiatric diagnoses including autism spectrum disorder, schizophrenia spectrum disorders, other psychotic disorders, or mood disorders with psychotic features, based on clinical interview or the K-SADS-PL; (4) individuals who had been prescribed any psychotropic medications, including psychostimulants, within the month prior to their first visit and Q-EEG measurement; (5) individuals with severe congenital diseases under ongoing treatment; (6) individuals with a history of convulsive disorders; (7) individuals with a history of hearing impairment or visual impairment.

This study was based on retrospective chart review. It received ethical approval from the Samsung Medical Center Institutional Review Board (IRB No. SMC 2022-09-132). The study for healthy control group was performed independently. It also received ethical approval from the Samsung Medical Center Institutional Review Board (IRB No. SMC 2018-07-106).

### Cognitive measures, ADHD symptom evaluation, and behavioral measures

All participants were assessed with the Korean version of the ADHD Rating Scale (K-ARS), the Korean version of Wechsler Intelligence Scale for Children 4th edition (K-WISC-IV), the Korean Vineland Adaptive Behavior Scales-2nd edition (K-Vineland-II), Conner’s Continuous Performance Test (CCPT), and Korea-Child Behavior Checklist (K-CBCL).

Furthermore, all participants underwent clinical psychiatric interviews conducted by psychiatrists to diagnose ADHD, assess ADHD symptoms, and determine the presence of psychiatric comorbidities, including anxiety disorders and other psychiatric disorders. The psychiatric diagnoses were subsequently confirmed by clinical psychologists using the K-SADS.

The K-ARS, a scale composed of 18 items (9 items for assessing inattention and another 9 items for assessing hyperactivity) was conducted to assess the severity of ADHD symptoms. Participants rated the presence of symptoms over the past week on a scale ranging from 0 (not at all) to 3 (very much) for each item. For this study, the parent-report version of the K-ARS completed on the first visit was employed.

The K-WISC-IV was conducted to evaluate the intelligence of the children in the study. The K-WISC-IV is the Korean version of the WISC-IV (17), which is a four-factor intelligence battery for children aged between 6 and 16 years of age. This battery has 10 core subtests that contribute to the formation of four psychometrically validated factor scores: Verbal Comprehension Index (VCI), Perceptual Reasoning index (PRI), Working Memory Index (WMI), and Processing Speed Index (PSI). Additionally, all 10 subtests is combined to form a full-scale IQ score (FSIQ).

In addition to assessing overall intelligence, the CCPT was administered to evaluate the sustained and selective attention of the participants. The CCPT is a computerized continuous performance task (18) that involved the presentation of 360 letters on the screen, one at a time, for approximately 250 ms each. The task consisted of 360 trials divided into 18 consecutive blocks of 20 trials, with each block using one of three interstimulus intervals (ISI) (1, 2, or 4 s). The Total duration for the task administration was 14 min.

The CCPT provided T-scores for omission and commission errors, signal detection parameters, and reaction time. The average speed of all target responses for the entire test (Hit Reaction Time) served as an overall response time measure. Variability dependent measures included the standard error of Hit Reaction Time (Hit Reaction Time SE); a measures of “within-respondent” variability that could compare the variability of 18 time blocks to the overall variability, Hit reaction time SE (Variability); change in reaction time across the duration of the test (Hit Reaction Time Block Change) and its associated standard error (Hit SE Block Change); the change in mean reaction time for different ISIs (interstimulus intervals) of 1, 2, and 4 s (Hit Reaction Time ISI change) and its associated standard error (Hit SE ISI Change).

The CCPT also provided an indicator of unusual patterns of responding, such as unusually slow, random, or anticipatory responses, or repeated responses without consideration of the stimuli or task requirements (Perseveerations). Accuracy-dependent measures included the Omission error (failure to respond to target letters), Commission error (response to non-target letters), and detectability (d’), which measured the overall discriminative power to differentiate between signal and noise distributions. High T-scores indicated poor performance for all items except detectability (d’).

The K-Vineland-II was administered to evaluate the overall adaptation level of participants. The Vineland-II is designed to assess the daily functioning of an individuals, including both those who are typically developing and those with disabilities, within the age range from preschool to early adulthood. Three domains of measurement–Communication, Daily Living Skills, and Socialization Skills- represent various dimensions of adaptive behavior. For each domain, age-matched T-scores were calculated and an overall adaptive behavior composite score was generated by combining the scores across the domains. In each domain, a high T-score indicates better adaptive functioning.

The CBCL was administered to assess a wide range of childhood behaviors. The CBCL was completed by the main caregiver of each participant to assess behavior of participants in the past 6 months. The items on the CBCL measured specific emotional and behavioral problems on a 3-point Likert scale (0 = “Not True” to 2 = “Very True or Often True”). The CBCL consisted of two empirically derived broadband scales and eight syndrome scales (19). The broadband Internalizing Domain measured emotional problems and included three syndrome scales: Anxious/Depressed, Withdrawn/Depressed, and Somatic Complaints. The Broadband Externalizing Domain measured behavioral problems and included the Rule Breaking Behavior and Aggressive Behavior syndrome Sales. Three other syndrome scales (Social Problems, Thought Problems, and Attention Problems) did not belong to either internalizing or externalizing domain. The Total Problems scale provided an overall measure of impairment and was derived from the sum of the raw scores of all eight syndrome scales and a group of 17 Other Problems items that did not belong to any specific syndrome scale. The results are presented as behavioral problem T-scores, with a high T-score meaning more behavioral problems.

For all participants, diagnostic, behavioral, cognitive, and EEG data were collected prior to the initiation of medication. The results of the aforementioned assessments were presented and utilized for further analyses as age-matched T scores.

### EEG measures

Electroencephalography recording was conducted using a 62-channel digital electroencephalography device (62-ch SynAmps 2 Neuroscan system, Compumedics, Charlotte, NC, USA). Nineteen surface electrodes were positioned according to the international 10/20 location system (FP1, FP2, F3, F4, F7, F8, Fz, T3, T4, C3, C4, Cz, T5, T6, P3, P4, Pz, O1, and O2). All electrodes were referenced to linked ears, and each electrode was silver-plated. Impedance was below 5 Ohms. Each participant was asked to keep awake with eyes closed in a quiet room with sound insulation. EEG data were recorded for approximately 5 min, and experienced technicians examined the data to exclude any artifacts. The recorded EEG data were analyzed using a Q-EEG analysis software (NeuroGuide, Applied Neuroscience, Inc., Largo, FL, USA). The Continuous EEG data were Fourier transformed and divided into four frequency bands for further analysis: delta (1–4 Hz), theta (4–8 Hz), alpha (8–12 Hz), and beta (12–25 Hz). The power spectrum for each frequency band was calculated. The relative power (the ratio of each frequency band to the total) and theta/beta power ratio were calculated based on the absolute power values. Additionally, further analysis of the beta frequency band involved dividing it into sub-band by dividing bands into beta 1 (12.0–15.0 Hz), beta 2 (15.0–18.0 Hz), beta 3 (18.0–25.0 Hz), and high beta (25.0–30.0 Hz).

### Data analysis plan

Group differences on demographic, cognitive, and behavioral characteristics among ADHD-Only, ADHD-ANX, and HC groups were assessed by ANOVA (Analysis of Variance) for continuous dependent variables and chi-squared analysis for dichotomous variables (sex and comorbidity pattern).

In order to compare Q-EEG characteristics of participants according to presence or absence of ADHD and comorbid anxiety, intergroup comparisons for each location and frequency bands were performed through ANCOVA (Analysis of Covariance) using age, sex, and FSIQ as covariates considering their widely known impact on EEGs (20, 21). Relative power values from 19 electrodes were averaged for three regions in order to reduce the number of variables for statistical comparisons. These three regions were frontal (FP1, FP2, F3, F4, F7, F8, Fz), central (T3, T4, C3, C4, Cz), and posterior (T5,T6, P3, P4, O1, and O2). These regions were determined by principal component analysis with varimax rotation method.

Cluster analysis was conducted on participants with ADHD (ADHD-Only groups and ADHD-ANX groups) to explore the contribution of comorbid anxiety disorder to the heterogeneity of ADHD Q-EEG characteristics. Participants in these two groups were hierarchically clustered by Ward’s method, using the squared Euclidean distance to measure differences between subjects. The variables used in the cluster analysis were population-based age-matched z-score provided by the Neuroguide software of relative power estimates for 19 electrode locations of each frequency band. The z-scores standardized the contribution of each variable while accounting for the impact of age. Following the clustering process, chi-squared analysis was performed to examine difference in the ratios of subjects diagnosed with anxiety disorder within subgroups obtained as a result of cluster analysis. Statistical analyses (ANOVA) were conducted to compare clinical measures across subgroups. Additionally, intergroup comparisons (ANCOVA) were performed, considering age and FSIQ as covariates, to assess the differences between each subgroup and the control or overall ADHD group. A significance level of 0.05 (*p* < 0.05) was used for statistical significance in this study. If there were significant differences between groups, *post hoc* analyses with Bonferroni correction were conducted. Bonferroni correction were also conducted for multiple comparisons of behavioral and cognitive measures. In these cases, *p*-values are indicated as adjusted values.

## Results

### Demographic characteristics

A total of 209 children (179 males and 30 females) were included in the analysis. Out of the initial 185 children who met the inclusion criteria, 19 children were excluded based on the exclusion criteria, As a result, the analysis included 166 children with ADHD. The ADHD-Only group consisted of 141 children (126 males and 15 females). The ADHD-ANX group consisted of 25 children (20 males and 5 females). The specific diagnoses were as follows: 4 children with social anxiety disorder, 2 with generalized anxiety disorder, 3 with separation anxiety disorder, 1 with specific phobia, and 16 with other specified anxiety disorder. The HC group consisted of 43 children (33 males and 10 females). There were no significant differences in age or sex between ADHD-Only, ADHD-ANX, and HC groups. Detailed demographic characteristics of each group are presented in Table 1.

**TABLE 1:** Demographic characteristics of participants.

	ADHD-Only	ADHD-ANX	HC	statistics (*p*-value)	*post hoc*
*n*	141	25	43	–	–
Age	7.96 (1.77)	8.16 (2.10)	8.65 (1.40)	0.08	–
Sex (M:F)	126:15	20:5	33:10	0.08	–
Comorbidity [*n*, (%)]					
No comorbidity	96 (68.1%)	15 (60.0%)	43 (100.0%)	0.43	–
Tic/Tourette disorder	18 (12.8%)	5 (20.0%)	–	0.33	–
ODD*	15 (10.6%)	3 (12.0%)	–	0.84	–
Etc.	12 (8.5%)	2 (8.0%)	–	0.93	–

*Oppositional defiant disorder.

### Cognitive and behavioral measures

The mean K-ARS score was statistically significantly higher in the ADHD-Only group (mean ± standard deviation [SD], 24.87 ± 9.20) and the ADHD-ANX group (22.12 ± 8.90) compared to the HC group (4.81 ± 3.55). However, It was not significantly different between the ADHD-Only group and the ADHD-ANX group.

Regarding the K-WISC-IV, group differences were observed in FSIQ and all subscales including VCI, PRI, WMI, and PSI (Table 2). However, There were no significant differences in the items of K-WISC-IV between the ADHD-Only group and the ADHD-ANX group.

**TABLE 2:** Cognitive and behavioral measures of participants.

	ADHD-only	ADHD-ANX	Control	Statistics (adjusted *p*-value)	*post hoc*
**K_ARS**	24.87 (9.20)	22.12 (8.90)	4.81 (3.55)	**<0.01**	1 > 3, 2 > 3
inattention	13.69 (5.13)	12.52 (5.36)	3.09 (2.51)	**<0.01**	1 > 3, 2 > 3
hyperactivity	11.18 (5.24)	9.6 (5.10)	1.74 (1.45)	**<0.01**	1 > 3, 2 > 3
**FSIQ**	94.67 (15.47)	94.08 (11.16)	108.93 (13.23)	**<0.01**	1 > 3, 2 > 3
VCI	99.49 (13.81)	100.6 (12.42)	106.81 (11.72)	**0.04**	3 > 1
PRI	100.8 (16.61)	99.28 (15.26)	112 (13.77)	**0.01**	3 > 1, 3 > 2
WMI	93.78 (15.22)	91.64 (14.35)	107.65 (13.23)	**<0.01**	3 > 1, 3 > 2
PSI	88.15 (15.90)	89.68 (14.09)	99.77 (14.03)	**<0.01**	3 > 1, 3 > 2
**CPPT**					
d’	56.77 (7.87)	59.96 (9.06)	49.77 (10.00)	**<0.01**	1 > 3, 2 > 3
omission	63.02 (16.13)	68.2 (17.60)	52.91 (13.17)	**<0.01**	1 > 3, 2 > 3
commission	51.11 (8.88)	51.8 (11.69)	45.44 (9.74)	**0.02**	1 > 3, 2 > 3
perseveration	58.97 (14.69)	58.76 (15.62)	52.7 (10.66)	0.33	-
HRT	57.51 (10.16)	56.92 (10.39)	57.28 (11.24)	0.34	-
HRT SE	61.36 (13.12)	63.08 (16.56)	53.47 (10.00)	**<0.01**	1 > 3, 2 > 3
Variability	58.2 (11.81)	57.05 (11.19)	50.59 (8.16)	**<0.01**	1 > 3
HRT BC	52.04 (13.20)	53 (18.37)	46.19 (10.09)	0.32	-
HRT ISI C	58.7 (13.79)	60.5 (14.54)	53.76 (11.18)	0.07	-

Data are presented as mean (standard deviation) unless otherwise indicated. The numbers highlighted in bold indicate statistical significance.

In terms of the CPT, the HC group demonstrated significantly lower scores (indicating better attention performance) than the other two groups in d,’ omission errors, commission errors, HRT SE and Variability. No significant differences in any CPT items were observed between the ADHD-Only group and the ADHD-ANX group.

Regarding the K-Vineland-II, the HC group exhibited significantly higher scores (indicating better adaptive functioning) than the other two groups at all times. No significant group differences were observed in any item of K-Vineland-II between the ADHD-Only group and the ADHD-ANX group.

On the K-CBCL, the HC group showed significantly lower scores (indicating fewer behavioral problems) than the other two ADHD groups at all time. The ADHD-ANX group had significantly higher scores in Anxious/Depressed domain (ADHD-only: 60.36 ± 8.69, ADHD-ANX: 66.33 ± 8.86, *p* < 0.01), and thought problem domain (ADHD-only: 61.71 ± 7.84, ADHD-ANX: 66.63 ± 6.36, *p* < 0.01) (Table 2).

### EEG spectral analysis for relative power by band

**FIGURE 1:**
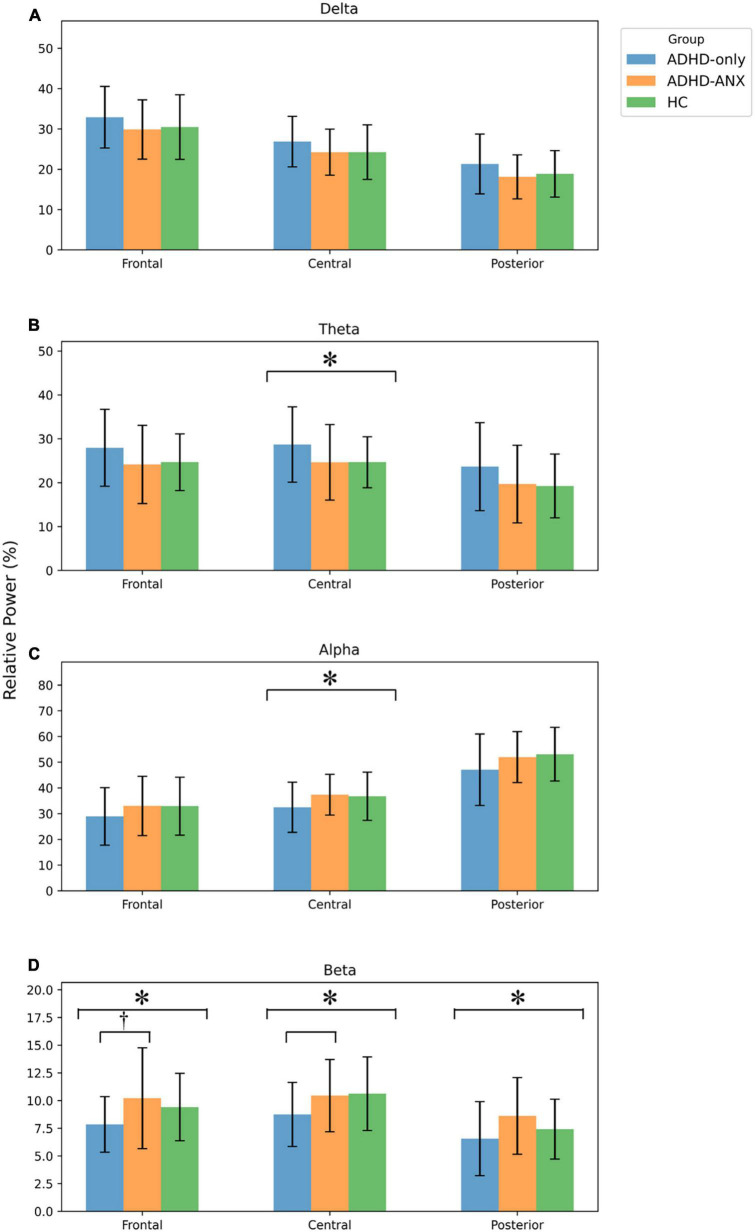
EEG relative power of ADHD-only, ADHD-ANX, and healthy control groups in frontal, central, and posterior regions. Panel **(A)** shows relative power of delta frequency band in frontal, central, and posterior regions. Panel **(B)** shows relative power of theta frequency band in frontal, central, and posterior regions. Panel **(C)** shows relative power of alpha frequency band in frontal, central, and posterior regions. Panel **(D)** shows relative power of beta frequency band in frontal, central, and posterior regions. *, significant (*p* < 0.05) group difference in ANCOVA, ^†^ significant group difference in *post hoc* analyses.

Group differences were observed in the theta band in the central region and the beta band in the frontal, central, and posterior regions. In the *post hoc* analyses, the ADHD-ANX group showed significantly higher beta power values than the ADHD-Only group in all regions. However, There were no significant group differences in beta bands between the HC group and the other two groups in any of the regions (Figure 1 and Table 3).

**TABLE 3:** Q-EEG relative power value of each frequency band.

Relative_Power (%)	ADHD-only	ADHD-ANX	Control	Statistics (*p*-value)	*post hoc*
F_Delta	32.92 (7.63)	29.87 (7.36)	30.47 (8.02)	0.14	–
C_Delta	26.87 (6.28)	24.25 (5.71)	24.26 (6.76)	0.10	–
P_Delta	21.33 (7.41)	18.12 (5.45)	18.86 (5.77)	0.08	–
F_Theta	27.94 (8.75)	24.16 (8.91)	24.66 (6.45)	0.11	–
C_Theta	28.71 (8.57)	24.64 (8.59)	24.66 (5.80)	**0.04**	–
P_Theta	23.67 (10.03)	19.7 (8.85)	19.26 (7.27)	0.10	–
F_Alpha	28.93 (11.17)	33 (11.54)	32.9 (11.25)	0.19	–
C_Alpha	32.48 (9.74)	37.38 (7.93)	36.75 (9.37)	**0.04**	–
P_Alpha	47.06 (13.9)	51.97 (9.91)	53.1 (10.45)	0.13	–
F_Beta	7.85 (2.51)	10.21 (4.55)	9.42 (3.04)	**<0.01**	1 < 2
C_Beta	8.75 (2.89)	10.45 (3.26)	10.62 (3.32)	**0.01**	1 < 2
P_Beta	6.57 (3.34)	8.62 (3.46)	7.42 (2.7)	**0.02**	1 < 2
F_TBR	4.14 (2.39)	2.95 (1.86)	3.06 (1.64)	**0.03**	1 > 2
C_TBR	4.21 (2.41)	2.98 (2.01)	2.92 (1.35)	**0.01**	1 > 2
P_TBR	4.65 (3.51)	2.9 (2.16)	3.23 (2.28)	**0.03**	1 > 2

Data are presented as mean (standard deviation) unless otherwise indicated.

In additional analysis, the beta frequency range was further subdivided into beta 1, beta 2, beta 3, and high beta sub-bands.

The results revealed that the ADHD-ANX group had significantly higher values than the ADHD-Only group in the frontal beta 1, central beta 1, posterior beta 2, frontal beta 3, central beta 3, and posterior beta 3 bands.

Additionally, the ADHD-Only group had significantly lower values than the HC group in frontal beta 3 and central beta 3 bands (Supplementary Table 1). The numbers highlighted in bold indicate statistical significance.

**FIGURE 2:**
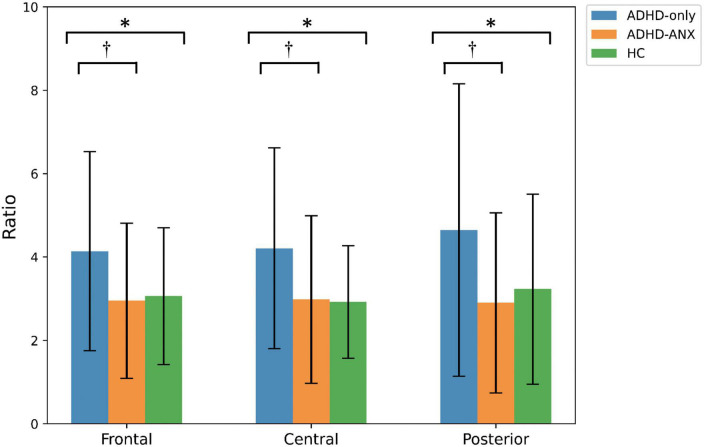
Theta to beta ratio of ADHD-only, ADHD-ANX, and healthy control groups in frontal, central, and posterior regions. *, significant (*p* < 0.05) group difference in ANCOVA; ^†^, significant group difference in *post hoc* analyses. By hierarchical clustering analysis based on Q-EEG relative power values, participants of the ADHD-Only group and the ADHD-ANX group were divided into four clusters. Cluster 1 consisted of 44 children (26.5% of children with ADHD), including 40 from the ADHD-Only group (28.4% of children in the ADHD-only group) and four from the ADHD-ANX group (16.0% of children in the ADHD-ANX group). Cluster 2 consisted of 72 children (43.4% of children with ADHD), including 60 from the ADHD-Only group (42.6% of children in the ADHD-only group), and 12 from the ADHD-ANX group (48.0% of children in the ADHD-ANX group). Cluster 3 consisted of 39 children (23.5% of children with ADHD), including 35 from the ADHD-Only group (28.4% of children in the ADHD-only group) and 4 from the ADHD-ANX group (16.0% of children in the ADHD-ANX group). Cluster 4 consisted of 11 children (6.6% of children with ADHD), including 6 from the ADHD-Only group (4.3% of children in the ADHD-only group), and 5 from the ADHD-ANX group (20.0% of children in the ADHD-ANX group). The ratios of psychiatric comorbidities showed statistically significant differences between clusters (*χ*^2^ = 8.43, *p* = 0.04). Furthermore, there were statistically significant difference in the ratios of children with comorbid anxiety disorder across each cluster. (*χ*^2^ = 10.02, *p* = 0.02) (Table 4).

For theta/beta ratio, the ADHD-Only group had significantly higher values than the ADHD-ANX group in the frontal, central, and posterior regions. However, there were no significant differences between the HC group and either the ADHD-Only group or the ADHD-ANX group (Figure 2 and Table 3).

**TABLE 4:** Distribution of ADHD participants in cluster analysis.

	Cluster 1	Cluster 2	Cluster 3	Cluster 4	*P*-value
**N (% in total ADHD patients)**	44 (26.5%)	72 (43.4%)	39 (23.5%)	11 (6.6%)	–
Comorbidities–*n* (% in each cluster)	21 (47.7%)	39 (54.2%)	12 (30.8%)	8 (72.7%)	**0.04**
Anxiety disorder (ADHD-ANX) –*n* (% in each cluster)	4 (9.1%)	12 (16.7%)	4 (10.3%)	5 (45.5%)	**0.02**
Tic disorder–*n* (% in each cluster)	10 (30.3%)	9 (10.0%)	4 (10.3%)	0 (0%)	0.16
ODD–*n* (% in each cluster)	4 (9.1%)	10 (13.9%)	1 (2.6%)	3 (27.3%)	0.08
Etc–*n* (% in each cluster)	3 (6.8%)	8 (29.6%)	3 (7.7%)	0 (0%)	0.60

No significant group differences were observed in K-ARS, K-WISC-IV, CBCL and K-Vineland-II.

However, in the CPT, there were group differences in omission and HRT SE (Hit Reaction Time standard error) among the clusters.

Nevertheless, the *post hoc* analyses did not reveal any significant group differences between specific clusters.

In comparison to the total participants with ADHD, cluster 1 exhibited increased theta, decreased alpha, decreased beta, and increased TBR. cluster 2 showed decreased delta, decreased theta, and increased alpha. cluster 3 showed increased delta, decreased frontal alpha, increased posterior beta, and decreased TBR.

**FIGURE 3:**
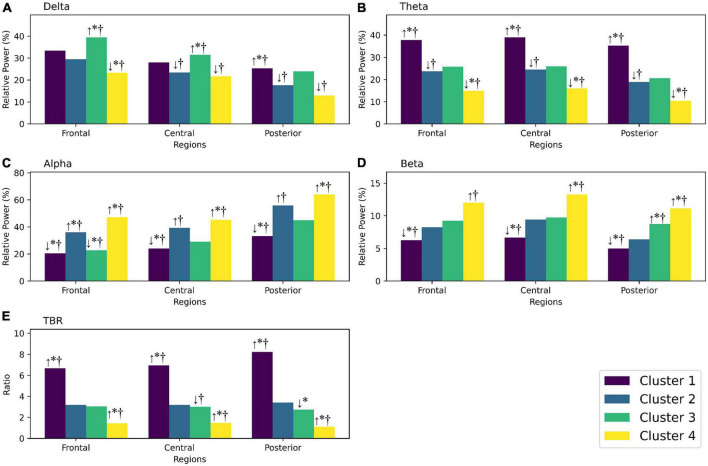
**(A)** Shows the relative power of the delta frequency band in frontal, central, and posterior regions for each cluster. **(B)** Shows the relative power of the theta frequency band for each cluster. **(C)** Shows the relative power of the alpha frequency band for each cluster. **(D)** Shows the relative power of the beta frequency band for each cluster. **(E)** Shows the theta/beta ratio for each cluster. If there are any statistically significant differences, they are indicated by an arrow showing whether the value is higher or lower than the reference group (the control group or the total ADHD group). *Significantly different (*p* < 0.05) from the control group; ^†^significantly different from the total ADHD group; ↑, higher mean value than the reference group; ↓, lower mean value than the reference group.

Finally, cluster 4 demonstrated decreased delta, decreased theta, increased alpha, increased beta and decreased TBR (Figure 3 and Table 5).

In comparison to the participants from the HC group, cluster 1 showed increased theta, decreased alpha, decreased beta, and increased TBR.

Cluster 2 exhibited increased frontal alpha. cluster 3 showed increased delta and decreased frontal alpha.

Lastly, cluster 4 displayed decreased frontal delta, decreased theta, increased alpha, increased beta, and decreased TBR (Figure 3 and Table 5). The numbers highlighted in bold indicate statistical significance.

**TABLE 5:** Q-EEG relative power values of each cluster.

Relative power (%)	Cluster 1	Cluster 2	Cluster 3	Cluster 4
D_frontal	33.42	29.49	39.43↑*^†^	23.31↓*^†^
D_central	28.07	23.48↓^†^	31.56↑*^†^	21.65↓^†^
D_posterior	25.38↑*^†^	17.59↓^†^	23.98	12.9↓^†^
T_frontal	37.84↑ *^†^	23.75↓^†^	25.76	14.89↓*^†^
T_central	38.97↑*^†^	24.45↓^†^	25.97	16↓*^†^
T_posterior	35.32↑*^†^	18.9↓^†^	20.57	10.34↓*^†^
A__frontal	20.45↓*^†^	36.12↑*^†^	22.69↓*^†^	47.14↑*^†^
A_central	23.96↓*^†^	39.25↑^†^	29.13	45.28↑*^†^
A_posteror	33.22↓*^†^	55.82↑^†^	44.9	63.9↑*^†^
B_frontal	6.27↓*^†^	8.25	9.24	12↑^†^
B_central	6.65↓*	9.4	9.75	13.25↑*^†^
B_posteror	4.99↓*^†^	6.38	8.73↑*	11.16↑*^†^
TBR_frontal	6.68↑*^†^	3.18	3.04	1.44↓*^†^
TBR_central	6.95↑*^†^	3.18	3.01↓^†^	1.47↓*^†^
TBR_posterior	8.24↑*^†^	3.42	2.73↓*	1.12↓*^†^

If there are any statistically significant differences, They are indicated by an arrow showing whether the value is higher or lower than the reference group (the control group or the total ADHD group).

*, significantly different (*p* < 0.05) from the control group; ^†^, significantly different from the total ADHD group; ↑, higher mean value than the reference group; ↓, lower mean value than the reference group.

## Discussion

Many studies have reported findings that children with ADHD exhibit distinct EEG characteristics (9–11), and there have been ongoing attempts to utilize Q-EEG for differential diagnosis or treatment outcome evaluation of ADHD. However, studies exploring Q-EEG differences based on a comorbidities with anxiety disorder in ADHD have been limited.

In this study, participants with ADHD were classified by the presence or absence of comorbid anxiety disorder, and their Q-EEG characteristics were compared. The results revealed that the ADHD-Only group and the ADHD-ANX group did not show significant group differences in clinical characteristics, except for the anxiety/depression item or the thought process item in CBCL. However, group differences were observed in Q-EEG. Specifically, The ADHD-ANX group showed significantly higher beta values but lower TBR compared to the ADHD-Only group. On the other hand, there were no significant group differences in Q-EEG between the ADHD-Only group and the HC group.

In the results of additional clustering analysis, Cluster 1 could be identified as a group with widely known ADHD characteristics, such as higher theta, lower beta, and increased TBR. However, cluster 1 accounted for only 26.5% of ADHD participants. Cluster 2, comprising 43.4% of the participants with ADHD, did not show significant differences in Q-EEG characteristics compared to healthy controls, except for frontal alpha. Similarly, participants in cluster 3, accounting for 23.5% of children with ADHD, did not show significant differences, except for frontal delta, central delta, and frontal alpha, when compared to healthy controls. Cluster 4, characterized by excessive beta and decreased TBR compared to the control group and total ADHD group, showed high comorbidity (7 of 11 participants), with a considerable number of them also exhibiting comorbid anxiety disorder (5 of 11 participants).

The results of this study revealed that participants with ADHD did not show a significant increase in theta, decrease in beta, or increase in TBR compared to the control group. Only a subgroup, cluster 1, had a high TBR compared to the control group. These results were different from a previous study reporting that increased theta, decreased beta, and increased TBR were commonly observed in ADHD relative to normal controls (9).

On the other hand, a meta-analysis suggested that the diagnostic and prognostic value of TBR is limited to a subgroup of ADHD because of the heterogeneity of Q-EEG in ADHD (11), which aligns with the results of this study. Some researchers have identified a distinct subgroup with increased alpha and beta waves, accompanied by a higher level of internalizing behaviors and emotion dysregulation (13). These findings are consistent with the results of the current study, where the ADHD-ANX group exhibited higher beta values in all regions, lower theta values in central region, and lower TBR in all regions compared to the ADHD-Only group. Cluster 4, which showed significantly higher alpha and beta values than the HC group, also had significantly higher rate of comorbid anxiety.

Previous studies have suggested that ADHD children with a comorbidity for anxiety disorder exhibit different cognitive or behavioral characteristics compared to those without a comorbidity for anxiety disorder, such as lower impulsivity and worse working memory (4). However, in this study, no group differences in cognitive or behavioral measures, including K-ARS, K-WISC-IV, K-Vine l and-II, or CPT, were observed between ADHD children with anxiety and those without anxiety. Interestingly, EEG, as an electrophysiological measure, showed better discrimination between children with a comorbidity for anxiety disorder and those without a comorbidity for anxiety disorder, compared to behavioral and cognitive measures.

How can beta activity be increased in ADHD children with a comorbidity for anxiety disorder? The association between beta excess and anxiety has been reported repeatedly (14, 15, 22). Similarly, In this study, ADHD children with a comorbidity for anxiety disorder exhibited higher relative power in beta frequency compared to ADHD children without anxiety. These results were are consistent to previous studies, suggesting that similar biological mechanisms might contribute to beta excess in ADHD children with a comorbidity for anxiety disorder.

Some research studies have proposed that comorbid anxiety can moderate certain ADHD symptoms, particularly externalizing behaviors such as aggression. A 10-year longitudinal study reported that anxiety has a protective effect against proactive and reactive aggression of ADHD patients (23). If the attenuation hypothesis is true, ADHD symptoms of children with a comorbid anxiety disorder might have been underestimated or not diagnosed early. Therefore, when using Q-EEG as an supplementary tool for ADHD diagnosis, it is necessary to understand their unique Q-EEG characteristics, in addition to recognizing the risk of ADHD symptoms being underestimated based on results of this study.

Excessive beta activity in ADHD with a comorbidity for anxiety disorder might also have implications for the selection and use of medication for ADHD. It has been reported that the use of methylphenidate can increase frontal beta activity, and there is a correlation between beta increase and clinical response to medication (24). On the other hand, there are several reports showing that the effect of methylphenidate decreases in ADHD children with comorbid anxiety (7, 25). Some studies have suggested that atomoxetine should be considered for the treatment of ADHD in youth who have ADHD with comorbid anxiety disorder (26). The excessive beta activity observed in drug- naïve ADHD children with comorbid anxiety disorder in this study could provide an explanation for the previously reported limited medication response.

In this study, the majority of psychiatric comorbidities (such as Oppositional defiant disorder and tic disorders) were included except for autism spectrum disorder and intellectual disability, which might have limitations to perform tests. Considering the high psychiatric comorbidity of ADHD, this research setting reflected clinical reality better. In addition, the effect of psychotropic medication on Q-EEG was excluded by including only drug-naïve subjects.

This study should be understood in the context of the following limitations. Firstly, because subjects in the control group were recruited independently, their baseline characteristics were slightly different from those in the patient group. However, by correcting FSIQ, age, and sex, the impact of differences was minimized. Secondly, due to the limited number of participants, anxiety disorders were analyzed as a single category without further subdivision. Consequently, the differences between individual anxiety disorders could not be revealed. Thirdly, this study did not compare children with anxiety disorder but without ADHD. Typically, children who primarily present with anxiety symptoms do not undergo Q-EEG examinations during hospital visits. Since this study was conducted retrospectively, it was challenging to investigate this specific group of children.

This study classified children with ADHD based on the presence or absence of a comorbidity for anxiety disorder and compared their Q-EEG values. Children with both ADHD and comorbid anxiety disorder exhibited lower theta power in the central region, higher beta power across all regions and lower TBR across all regions compared to those without comorbid anxiety disorder. In the additional cluster analysis, children with ADHD were classified into four clusters with distinct Q-EEG characteristics, and it was observed that these clusters exhibited differences in the rates of psychiatric comorbidities among the children within them. Specifically, The ratios of children with comorbidities for anxiety disorder within each cluster were significantly different. If studies with prospective design are conducted on these children with both ADHD and anxiety disorder, the relationship between anxiety and ADHD may be further revealed.

## Data availability statement

The original contributions presented in this study are included in this article/Supplementary material, further inquiries can be directed to the corresponding author.

## Ethics statement

The studies involving human participants were reviewed and approved by the Institutional Review Board, Samsung Medical Center. Written informed consent from the participants’ legal guardian/next of kin was not required to participate in this study in accordance with the national legislation and the institutional requirements.

## Author contributions

CJ and Y-SJ conceived the study. CJ and SO designed the research methodology. CJ organized the patient group data and wrote the initial draft of the manuscript in discussion with IS, YP, and EL. SO organized the control group data. HL and JL provided advice on the analysis method. Y-SJ supervised the entire process. All authors contributed to the manuscript and approved the submitted version.
