# Supplementary material for: The impact of comorbid anxiety on quantitative EEG heterogeneity in children with attention-deficit/hyperactivity disorder
Source: Front Psychiatry. 2023 Jul 12;14:1190713. doi: 10.3389/fpsyt.2023.1190713 (PMC10368871; doi:10.3389/fpsyt.2023.1190713)
Supplement: Supplementary file 1 [file Table_1.DOCX]

Table S1. Q-EEG relative power values of beta 1, beta 2, beta 3, and high beta band

| **Relative_Power** | ADHD-only | ADHD-ANX | Control | Statistics (p value) | Post hoc |
| --- | --- | --- | --- | --- | --- |
| F_High Beta | 1.18 | 1.47 | 1.38 | **0.04** | - |
| C_High Beta | 1.36 | 1.54 | 1.56 | 0.29 | - |
| P_High Beta | 0.64 | 0.76 | 0.66 | 0.35 | - |
| F_Beta1 | 2.91 | 3.72 | 3.37 | **0.01** | 1 < 2 |
| C_Beta1 | 3.23 | 4.01 | 4.02 | **0.02** | 1 < 2 |
| P_Beta1 | 2.9 | 3.95 | 3.39 | 0.09 | - |
| F_Beta2 | 1.85 | 2.17 | 2.01 | 0.06 | - |
| C_Beta2 | 2.15 | 2.38 | 2.36 | 0.30 | - |
| P_Beta2 | 1.58 | 1.9 | 1.62 | **0.04** | 1 < 2 |
| F_Beta3 | 3.09 | 4.31 | 4.03 | **<0.01** | 1 < 2, 1 < 3 |
| C_Beta3 | 3.38 | 4.07 | 4.23 | **0.01** | 1 < 3 |
| P_Beta3 | 2.09 | 2.77 | 2.39 | **<0.01** | 1 < 2 |
